# Co‐design to deliver service improvement: What does this mean and how can we do it? A qualitative study with upper gastrointestinal cancer patients and professionals

**DOI:** 10.1002/cnr2.1748

**Published:** 2022-11-08

**Authors:** Anna Haste, Linda Sharp, Richard Thomson, Sarah Sowden

**Affiliations:** ^1^ Population Health Sciences Institute Newcastle University Newcastle upon Tyne UK; ^2^ Centre for Applied Psychological Science, Department of Psychology, School of Social Sciences, Humanities and Law Teesside University Middlesbrough UK; ^3^ Newcastle University Centre for Cancer, Population Health Sciences Institute Newcastle University Newcastle upon Tyne UK

**Keywords:** cancer, co‐design, health care professionals, patients, service improvement

## Abstract

**Background:**

There is strategic objective to incorporate the principles and practice of co‐design into routine service development and improvement.

**Aim:**

The aim was to explore the concept and feasibility of service co‐design with patients and health professionals with regards to the upper gastrointestinal (UGI) cancer care pathway.

**Methods and Results:**

Qualitative telephone interviews and face‐to‐face focus groups in one region of England. Twenty patients completed interviews. Nine patients and ten professionals formed two focus groups. Patients were referred through the urgent (two week) GP referral route and were within six months of receiving their first treatment for an UGI cancer. Professionals were working as service planners and providers of the UGI cancer care pathway. Thematic analysis was undertaken. Six themes emerged: Responsibilities and expectations, Knowledge and understanding, Valuing patient input, Building relationships, Environment for co‐design activities, Impact and effectiveness. Based on the themes a checklist has been created to provide practical suggestions for both professionals and patients on approaching co‐design for service improvement.

**Conclusion:**

This study offers policy and practice partners a clearer understanding of co‐design and factors to consider when approaching co‐design in real life settings.

## 1 INTRODUCTION

Tailoring care to patient need and improving patient experience are frequently cited goals of healthcare systems internationally.^1^ Co‐design of services with professionals, patients and the public is heralded as a mechanism through which person‐centred care may be achieved. For example, co‐design, that is co‐creation of services with stakeholders, is recognised as one of the nine principles required for the successful implementation of the 2015 Cancer Strategy for England.^2^

Existing systematic reviews provide a synthesis of evidence on co‐design within healthcare. As a result of co‐design developments within health care services have been implemented.^3^ The implemented changes included: the production of new or improved sources of information for patients; simplifying appointment procedures; extending clinic opening times; improving transport to treatment units; improving access for people with disabilities; and a cultural change within organisations to involve patients. The strategic imperative to embed co‐design within service development is supported by evidence that undertaking co‐design leads to better outcomes and more person‐centred care, with another systematic review summarising evidence from 55 studies.^4^ Positive associations were presented between patient experience and self‐rated and objectively measured health outcomes including adherence to recommended clinical practice and medication; preventive care (such as health‐promoting behaviour, use of screening services and immunisation); and resource use (such as hospitalisation, length of stay and primary‐care visits).^4^

A study in pancreatic cancer identified that views of patients were often contrary to staff preconceptions of the desired care experience and illustrates how accessing patient views can identify specific problems and lead to real improvements for patients.^5^ Thus, the co‐design process can lead to increased understanding of different perspectives,^6^ resulting in broader potential changes to mind‐sets and ultimately behaviour, as well as healthcare staff reporting a greater sense of empowerment to make service changes.^7^ In addition, qualitative research has shown a link between staff wellbeing and patient experience, therefore it is not only patients who may benefit from such a shift.^8^

Co‐design has been applied in a range of clinical areas including cancer services. Previous successful studies using co‐design within cancer services in the UK and elsewhere suggest considerable potential for the application of this approach to redesign other elements of the cancer patient pathway.^5^, ^7^, ^9^, ^10^, ^11^, ^12^, ^13^ Some examples include, how and when information is provided to patients, explanations of the pathway and expectations of the timeframes involved, particularly in relation to appointments and results.

However, despite strategic proclamations of its critical importance and evidence supporting its effectiveness, what co‐design means, and how to achieve it, is far less clear. A variety of terminologies are used to describe patient involvement in service redesign: co‐production, participatory action research, co‐design, experience‐based co‐design and accelerated experienced based co‐design. At its most basic, co‐design represents a structured process for involving patients throughout all stages of quality improvement. The co‐design process captures the patient's lived experience, aims to understand these experiences and improve them.^14^ This has been characterised as involving six key phases: engage, plan, explore, develop, decide and change.^15^ A range of data collection techniques have been utilised within the co‐design process, including ethnographic observations, interviews, focus groups, questionnaires and documentary analysis involving patients, staff and family members as appropriate. However, most can be time and resource intensive and not necessarily tailored to local context.^16^

In 2000, in England, in part as a response to low cancer survival rates, a series of waiting time targets were established. These included a target of 14 days between urgent referral (usually from a GP) with symptoms suggesting cancer and first assessment by a specialist; this is known as the urgent cancer referral—or 2 week wait (TWW) pathway. In recent years, the NHS has struggled to meet these targets for UGI, and other cancers—a situation exacerbated by the pandemic.^17^, ^18^ Recent qualitative research has highlighted issues with UGI cancer patient experiences of the TWW pathway indicating it may be a clinical area where co‐design could be of value.^19^

It has been recommended that support is given to allow co‐design to become more widespread^10^ and there is a recognition that co‐design should become embedded into local routine practice. However, survival rates for upper GI cancers are low (5%–20%), 5 years after diagnosis.^20^ Therefore, in order for this to happen in cancer services, policy and practice partners require a better understanding of how to undertake co‐design in real life settings. With so many existing definitions co‐design remains an evolving concept.^21^ Therefore, research investigating how co‐design, with both health care professionals and patients, could be effectively incorporated within the cancer care pathways is vital to enable evidence to be translated into practice.

To date, no studies have been conducted within the UK using co‐design specifically with UGI cancer patients. In addition, whilst existing studies articulate some of the issues raised when undertaking co‐design (e.g., time constraints, power imbalances,^22^, ^23^, ^24^ the current literature fails to provide an adequate procedural understanding of implementing co‐design in practice). This study aimed to address this by: exploring the concept and feasibility of service co‐design of the UGI cancer care pathway with patients and professionals (commissioners and providers); identifying barriers and facilitators to undertaking co‐design in practice; and producing a practical ‘how to’ checklist, outlining preliminary and preparatory steps with practical implementation recommendations. The study research question is what barriers and facilitators do UGI patients and professionals identify in relation to co‐design in practice?

## 2 METHOD

### 2.1 Methodological approach

Two phases of data collection were undertaken. Firstly, qualitative semi‐structured telephone interviews were conducted, in 2017, with UGI patients. The majority of the topic guide for these interviews covered the patient experience of UGI cancer care pathway, the findings of which have been reported separately.^19^ The guide also included questions covering the concept of co‐design, asking interviewees how they felt about patients and professionals working together to improve services. Findings relevant to this line of enquiry are reported here.

Subsequently, focus groups were conducted to generate discussion and greater depth around issues relevant to co‐design. Two focus groups, conducted in 2017, one with patients and one with professionals (providers and commissioners) sought to identify barriers and facilitators to the adoption of co‐design. Each group lasted 90 min and was facilitated in a semi‐structured way, guided by PowerPoint presentations adjusted for each audience, in order to ensure the concept of co‐design was introduced and its feasibility fully explored. Focus groups encourage participants to play active roles and share their points of view,^25^ adding depth to the insights gained and maximising the opportunity for this work to inform the application of ‘co‐design’ in the realm of cancer (and indeed other services).

The study was carried out in accordance with the consolidated criteria for reporting qualitative research (COREQ)^26^ (COREQ checklist included within Supplementary information 1) and guidance on standards for reporting qualitative research findings.^27^

## 3 PARTICIPANT RECRUITMENT

### 3.1 Patients

For both the focus group and interviews, clinical nurse specialists (CNSs) identified eligible patients within their clinics and completed a referral form for the research team. Eligibility criteria required patients to be within 6 months of receiving their first treatment for an UGI cancer and be referred initially through the TWW pathway in a region of England. Only patients who had already taken part in an interview for the study were asked whether they would be interested in participating in a follow‐up focus group and hence recruited directly (full description of recruitment and interview details provided in a previously published paper).^19^

### 3.2 Professionals

Professionals were emailed an invitation to participate in the professional focus group via one of the NHS Cancer Alliance communications networks. Cancer Alliances bring together the key organisations in their area to coordinate cancer care.

### 3.3 Patient and public Involvement statement

A patient representative formed part of the project steering group (together with various other stakeholders), and provided feedback on all documentation, including suitability of the recruitment method for patients, the interview topic guide and the structure and content of the focus groups.

### 3.4 Ethical considerations

The study was approved by the Newcastle University Faculty of Medical Sciences Research Ethics Committee (ref 1272/13714/2017) and informed consent was obtained from all participants. The study was performed in accordance with the Declaration of Helsinki.

All participants provided written consent before the interviews and focus groups began and were assured of confidentiality. Participants were made aware that direct quotes in published materials would be anonymised. There were no financial or other incentives offered to participate.

### 3.5 Data collection

To ensure analytical rigour, the focus groups were facilitated by two female researchers (AH & SS) both with PhDs and previous experience in interviewing and facilitating focus groups and without any previous relationship with the research participants. The focus groups were audio‐recorded and transcribed verbatim. Recordings along with the researchers' field notes enhanced trustworthiness of the data analysis by supporting the credibility of findings. The transcripts were anonymised with unique participant IDs linking the participants' contributions to a password‐protected spreadsheet. Once transcription has occurred and been checked, recordings were deleted.

All interviews (except one, which was face‐to‐face at the interviewee's request) were conducted by one researcher (AH) via telephone, audio‐recorded and transcribed verbatim. Interviews lasted approximately 1 h.

### 3.6 Data analysis

The interviews and the two focus groups were initially analysed separately, with findings subsequently combined. Data analysis used inductive coding that allowed themes to emerge from the data without prior categorisation. Thematic analysis was used to identify themes and followed six phases: (1) Data Familiarisation; (2) Generating initial codes; (3) Searching for themes; (4) Reviewing themes; (5) Defining and naming themes; and (6) Producing the report.^28^ NVivo 11 software was used to facilitate coding and analysis of transcribed data. One researcher (AH) coded all interview transcripts, with a selection of the interviews (20%) then coded by a second researcher (SS). One researcher (AH) coded the two focus group transcripts. The coding framework was then discussed and agreed within the full research team. Data saturation was defined using the principle outlined by Francis et al (2009)^50^ ‘*Stopping criterion*—*After 10 interviews, when three further interviews have been conducted with no new themes emerging, we will define this as the point of data saturation*.’ (P1234). The thematic findings were developed into a draft checklist providing tips and advice on how to implement co‐design in healthcare in the future. This was refined through discussion with the wider project team and also the project steering group.

Where findings arose only in the interviews, or one particular focus group, this has been made clear in the results. Illustrative quotes are provided to support the findings.

## 4 RESULTS

For the patient interviews, 27 patients were referred to the research team; four declined and three could not be contacted. Twenty patients (74% of referrals) were interviewed. All interviewees were white British from across North East and Cumbria, with 19 men and one woman. The mean age was 66 years (range 48–77).

Nine of the interviewees (eight men and one woman) attended the patient focus group. The average age of patient was 68 years, ranging from 49 to 75.

Ten participants attended the professional focus group. This consisted of a range of professions connected to the UGI cancer care pathway (Head of Quality, Service Delivery Lead, Delivery Manager, Cancer Manager, two Clinical nurse specialists, Consultant surgeon, Consultant Radiologist, Nurse Endoscopist and a GP). The attendees also worked for a range of different organisations and areas across the North East and Cumbria.

## 5 FINDINGS

Six themes related to co‐design emerged from the analysis: responsibilities and expectations of involvement in co‐design, knowledge and understanding, valuing patient input, building relationships, environment for co‐design activities, and impact and effectiveness.

Theme 1 Responsibilities and expectations of involvement in co‐design.

One aspect of co‐design that emerged as important was the need for expectations to be outlined clearly for each person involved in the process; this related to each individual's responsibilities but also as the co‐design group as a collective. This was deemed necessary to allow people to be involved to levels that were suitable for them, for example, not adding more pressure to professionals' working schedule or overburdening patients undergoing or recovering from treatment.

‘*It depends which aspect you're looking at…you can only comment on the treatment if you're being treated…hospitals follow you up for five years…ten, which I think is incredible. But it's ten years, I don't know how often they'll want to see me or any others but I suppose you can give feedback as to how your years has progressed or your last six months have progressed but I don't know how relevant that would be to somebody who's currently in treatment*.’ (Patient focus group).

As illustrated by the quote above, expectations were also discussed in terms of the timeframes that would be necessary and appropriate in a process such as co‐design. It was generally acknowledged that patients should be involved in the process of improving services as well as professionals.

Participants suggested that neither the professional nor the patient view was more important, but rather that each was important in different ways and for different areas of potential improvement Both patients and professionals believed patients would be assets to a co‐design process. This finding was largely supported in the patient interviews. Patients described how working together could enable everyone to gain a better perspective of each other's experiences, that is, patients and health care professionals.

‘*You've also got to take some responsibility…for your actions. It's no good expecting the professional to do it all for you…I mean you've got to take some responsibility and try and help them*’. (Patient focus group).

‘*Well, having been there and done it and worn the t‐shirt, I could see both sides of the divide*’. (Patient interview 14).

‘*…it's a great idea because patients don't know or they can't know what the medical staff have to go through in order to facilitate their treatment. But there again, I think with a little bit of respect, the medical staff may not be 100% aware of what it's like to get yourself to a hospital on a particular date, at a particular time. I mean I don't know how aware they are. In my experience, I think they are aware of the problem but yes, I mean if patients were made more aware of what the medical staff have to do and if the medical staff are made more aware of what the patients have to do, that can only be a good thing. It can only be a good thing*’. (Patient interview 13).

However, not all patients interviewed expressed a desire to be involved in service development, instead expecting to this to be the domain of professionals only.

‘*To be honest, I think I would leave it to the professionals. Because, the job, I don't think if I was any of those professionals I would be wanting members of the public to interfere. And, that is personal view. Because, I couldn't see me, or any member of the public, in my opinion that could have done anything that would have made my stay there better*’. (Patient interview 4).

Theme 2 .Knowledge and understanding.

Having the knowledge to understand what is needed to improve a service was discussed within the focus groups and interviews. There were varied opinions of what being knowledgeable meant, for example, professionals may have the clinical knowledge, but patients can provide key information in relation to improving patient experience.

‘*…we would always say, “Well, we know what you need,” and the patients say, “Well, I know what I want.”…So there's a tension there and I do not think anyone contributor trumps the other, but there has to be an understanding. I think sometimes we do not do the understanding bit very well. In other words, we do not explain why we think this might be the right way to people who are making very reasonable contributions, and then they get disheartened*’. (Professional focus group).

A lack of understanding and ability, dependent on education or social position, were identified by interview respondents as potential barriers to patients getting involved, or being considered appropriate stakeholders, in the co‐design process.

‘*People see things differently…It's an awful thing to say but it does come down to social status and education…Your ability to assimilate information and rationalise it, is not universal. Therefore it would reflect on what sort of response you would get*’. (Patient interview 2).

Training and education were suggested as a way of preparing people to be involved in a process such as co‐design.

Theme 3 Valuing patient input.

It was acknowledged by professionals that the inclusion of patients can often be seen as meeting a requirement rather than being considered a valued and equal contribution.

‘*…we often say, “Let's have some patient representatives. Let's invite a couple of patients who are keen and everything, and there are twenty doctors or nurses sitting in a room with two patients. I think that's crackers. I actually think it should be equal numbers*’. (Professional focus group).

The quote illustrates that in order to truly get productive input from different stakeholders, for example, professional and patients together, representation within groups should be reassessed. Shifting the power balance within groups was seen as important to provide greater equity in opinions and inputs.

‘*I think you would have to understand what people are bringing to the table. You need someone who is actually going to challenge. I think there's certain medical staff…a lot of people would not dare ask them a question…Let alone challenge them. So, as I say, you need… if you are going to make progress with that, you need a certain level of understanding I think, and you need a certain level of willing to challenge people. You know, not just be wowed by the fact that someone is a Prof, or a specialist in their field*’. (Patient interview 24).

Theme 4 Building relationships.

Building relationships and trust within a group was deemed to be crucial to the co‐design process. In order to build relationships, it was felt that time was needed to learn about people and what they could bring to a group, rather than going straight into solving problems. This would allow participants to feel comfortable and encourage greater active participation in the long term.

‘*Most people have personal barriers anyway because that's the way we are as human beings. So most people aren't going to be completely open and honest with you at the first meeting. You have to build a relationship and when you're looking at getting information, sometimes that takes time*’. (Professional focus group).

‘*A patient might not have the confidence and there can be things like communication, and fortunately we're all right but certain groups of mental health issues or elderly struggle*’. (Patient interview 10).

Building relationships within a group can also create supportive environments in which participants may be more productive and honest. To aid this, feedback throughout the process was considered important, so that participants felt that their involvement and views were valued. This was seen as important to both explain why and what service changes had taken place but also as a method of explaining if input from a co‐design group had not been implemented. It was believed that providing this level of transparency to the process would embed trust and worth in the participants, whilst reducing the chance of participants becoming disheartened.

Theme 5 Environment for co‐design activities.

The environment in which co‐design occurs was discussed predominantly within the professional focus group. Participants spoke about how people tend to naturally stay within their traditional environments and require others to adapt (e.g., researchers conducting focus groups in a university building) but that this may not be conducive to successful co‐design.

‘*We give precious little attention to the actual environment in which we do these things. Even if it's about going to the environment within which those individuals are working and living, we might be more successful than the reverse of bringing them to our environment*’. (Professional focus group).

The format of ‘a meeting’ may be alien to some patients and may discourage them from participating in a co‐design progress.

‘*No. Me speaking personally, I wouldn't like to go into a meeting with various people, because that's just me. That's me as a person. I don't like anything like that*’. (Patient interview 25).

‘*All the number of times when using overhead projectors and somebody stands up in front and explains this is what they're going to do, it just alienates, in my view. It distances. Because time is precious, we want to be able to open those doors as quickly as possible because there will be time constraints*’. (Professional focus group).

Constraints, such as time and resources, were considered essential to consider prior to the co‐design process. Participants spoke about identifying clearly, from the outset, the expectations of participants and what is required for the process to be successful.

Theme 6 Impact and effectiveness.

The issue of how effective co‐design really is was identified predominantly within the patient focus group. Patients described providing feedback for healthcare they had previously received and pondered how much impact this had had.

‘*Well who are the important people to make the change? I mean we don't really know. I rather got the impression that some of the consultants really don't necessarily have sway there*’. (Patient focus group).

Patients felt that the impact of a co‐design process was dependent on higher level authorities/employees being open to incorporating change in a service.

‘*I think you tend to fill it [patient experience questionnaire] in based on your immediate… so I had one at the end of the last week I had a load of tests and,’ Fill this in. ‘I had no issues with it at all and perhaps you don't think back to little niggles, especially if you feel it was another hospital or somewhere else that was responsible for it*’ (Patient focus group).

Participants discussed how impact and effectiveness relied on when patients were approached in terms of disease course. This emerged as an important consideration, and echoes findings from theme one in relation to expectations within the co‐design process, as to how you involve participants, when and for how long.

### 5.1 Co‐design checklist

Table 1 shows the checklist of practical suggestions for both professionals and patients on how to approach co‐design for service improvement. The checklist includes a series of initial prompts and reflective questions that any group looking to undertake healthcare service redesign or development through a co‐design process would be advised to consider in terms of (a) preparing the ground work for the venture, (b) considering membership for the working group, (c) thinking through how any co‐design meetings/activity would be structured and (d) how feedback will be handled.

**TABLE 1:** Co‐design checklist

Preparation	
Have you considered…	Y/N
how to choose and engage a range of stakeholders chosen for the task? (e.g., what stage of the pathway should patients be for them to be right for the co‐design group? [under treatment/follow up])	
the power balance for the group? (e.g., number of patients and health care professional members included)	
whether family members or carers should be included in the process?	
how a co‐design group will be established?	
what budget is available? (e.g., travel, childcare or carer costs)	
the goals of the co‐design process?	
what timeframes are needed throughout the process? (e.g., end goals, intermediate tasks and repeated activities)	
what level of understanding is necessary (or needs providing)? (e.g., why the NHS/pathways need to change? How can changes be implemented?)	
Membership	
Have you considered…	
what are the expectations and responsibilities of the co‐design members? (e.g., should ‘contracts’ be signed?)	
what higher authority involvement is there to ensure timeframes are met and so changes can be enforced/implemented?	
how you will ensure patients and health care professionals work together from the start of the process?	
how different perspectives can be combined to best improve a service/pathway? (e.g., personal knowledge with technical or clinical knowledge)	
do members need access to training courses?	
Group activity	
Have you considered…	
what the ground rules of the meetings are? (e.g., terms of references)	
who will chair meetings? (e.g., using patient representatives to chair meetings)	
the environment that will be used for meetings? (e.g. public, NHS or university settings)	
allowing time in the process for members to build relationships and communicate effectively? (e.g., may need to focus on smaller or personal issues initially in order to progress to larger, significant pathway specific problems)	
using different communication styles? (e.g., technological advances—video calls)	
the need for preparation time as well as time for meetings?	
Feedback	
Have you considered…	
how will feedback be provided on members' considerations that have been put forward within the group?	
how feedback will be provided on end decisions and the rationale behind them?	
how long does knowledge stay relevant?	
how members will know they/the group have made a difference?	
using events as an opportunity for patient feedback? (e.g., annual events)	

## 6 DISCUSSION

This study has examined the concept and feasibility of cancer service co‐design with patients and professionals (both commissioner and providers). Six themes emerged from the analysis: responsibilities and expectations of involvement in co‐design, knowledge and understanding, valuing patient input, building relationships, environment for co‐design activities, and impact and effectiveness. The themes have enabled a deeper understanding of procedural considerations when implementing co‐design in practice and have been used to inform the development of a checklist of practical steps policy and practice partners may wish to consider when attempting to embark on co‐design in real life service settings. Importantly, the discussions in the focus groups and patient interviews led to the identification of important preparatory work and concrete practical considerations needed before any co‐design activity begins (e.g., deciding the members of the group and how the group will meet throughout the process and being clear about expectations of group members).

Some of the findings from our patient and professional focus groups compare to previous findings from INVOLVE's principles of co‐production^29^ and Think local act personal (TLAP)'s ‘Top ten tips for co‐production’.^30^ Common findings were the importance of equity of power, respecting and valuing the knowledge of all involved in the research, including all voices, reciprocity and building and maintaining relationships. Adding to these previous findings, the current study participants discussed the need to identify and embed vital stakeholders that could influence change in a service and identified the importance of high authority representation for buy‐in and to implement changes. In addition, providing feedback throughout a co‐design process was also deemed to be an essential requirement to promote clarity and transparency as to what could and could not be adopted and the rationale behind end decisions. These findings, together with our finding regarding the importance of concrete practical considerations, expand on the current literature base and illustrate how preparatory work and planning is essential to try and ensure the most effective process and end outcomes.

A remaining challenge of co‐design is the imbalance of power between patients and healthcare professionals; this has been highlighted as a potential barrier to the involvement of patients within health care improvement.^22^, ^23^, ^24^ These findings relate to the theme ‘environment for co‐design activities’ within the current study which identified the need to consider a variety of meeting settings and discussion styles to ensure patients feel welcomed and valued in the process. The need for a comfortable environment to enable members to open up about experiences was also highlighted. Easy access to the process and having a budget should allow for a range of patients and public to be involved, that is, travel costs, childcare, video calls or carer costs. It should be acknowledged that the data was collected in 2017. More options have emerged now as a result of the pandemic and it would be worth investigating how well options like tele or videoconferencing work in relation to undertaking successful co‐design.

Co‐design can create opportunities for patients and health care professionals to share their unique experiences and expertise to better understand each other's reality. In previous research, including in cancer, patients and health care professionals were able to develop a more comprehensive understanding of the pathway issues and worked together to implement realistic changes on behalf of the patients. As a result of the process patients felt ownership of their health care, motivated and empowered.^11^, ^13^ However, an identified barrier is the perception about the value patients could add to co‐design. It is worth noting that it is not only patients who have limited experience of co‐design; only a third of clinicians have knowledge of the term co‐design.^31^ Within this current study, it was identified that in order for co‐design to be effective all participants have to acknowledge [and accept] that a range of perspectives and knowledge need to be represented and—critically—truly valued, within the co‐design process. This is an important aspect as it enables a variety of opinions on how the overall pathway works not just a particular viewpoint.

As far as we are aware, this is the first study to explore the conceptualisation and feasibility of undertaking co‐design of service improvement for UGI cancer in the UK. The professional focus group included a range of professionals, with representatives from primary and secondary/tertiary care. These professionals were based across various NHS organisations within the North East and Cumbria. We were able to get not only professional but also patients' viewpoints in relation to co‐design. For patients, we were able to triangulate the findings from two different data collection methods (individual in‐depth interviews and a patient focus group). The consistency of thematic content uncovered using these two data collection processes lends weight to the findings identified from the data. A potential limitation is the inclusion of patients and professionals solely from the UGI cancer pathway, which may limit the generalisability of the findings. However, findings tended to relate to the use of co‐design in healthcare improvement generally rather than specifically within the UGI pathway. Clinical Nurse Specialists were used in the recruitment process; this may have resulted in selection of particular patients to be approached. However, participants expressed a range of views. Another limitation was the limited number of female participants, although UGI cancer is more common in males the proportion is still lower than expected and may have impacted on findings. The study lacked ethnic diversity among participants (all participants were white British). In part this reflects the ethnic diversity within the study area. However, previous research has identified ethnic minority cancer patients as reporting lower satisfaction, less positive experiences of care overall and less understanding of healthcare professionals.^32^ This has implications for ethnic inequalities in healthcare and the study misses the opportunity to uncover these. The professional and patient focus group were run separately, which is not entirely coherent with the concept of co‐design. However, our intention was to explore *attitudes towards* co‐design rather than undertaking it. From experience of working with professional and patient groups, and the relative power dynamics between the two, running separate focus groups for patients and professionals was deemed most appropriate to allow each group space to openly express their views and opinions around co‐design.

While there is strategic imperative to implement co‐design within healthcare for service improvement, co‐design is also increasingly being used in the development of behaviour change interventions for cancer survivors or to support survivors' self‐management of cancer and its consequences (see, e.g.,^33^, ^34^, ^35^, ^36^). Therefore these study findings also have wider applicability for intervention development research It has been recommended that understanding of the concepts of co‐design is an important aspect to avoid not only the implementation of ineffective or inefficient services or service solutions but also mistrust in health and social care services.^21^ Transferrable insights have been identified which are likely to be relevant to co‐design within other complex patient pathways (cancer and beyond), and these have been developed into a practical checklist. The checklist requires further research and dissemination to examine whether it is beneficial in aiding the preparatory stages of the co‐design process.

Previous research examining co‐design within health care settings usually provides limited detail on how this should be implemented and what the process should consist of. By providing a checklist of elements to consider when attempting to embark on co‐design activity, which is grounded in a thematic analysis of reported barriers and facilitators to co‐design, this study offers policy and practice partners a clearer understanding of how to implement co‐design in real life settings. This may assist them in fulfilling the strategic objective to incorporate the principles and practice of co‐design into routine service development and improvement.

## AUTHOR CONTRIBUTIONS

**Anna Haste:** Data curation (lead); formal analysis (lead); writing – original draft (lead); writing – review and editing (lead). **Linda Sharp:** Supervision (equal); writing – review and editing (equal). **Richard Thomson:** Supervision (equal); writing – review and editing (equal). **Sarah Sowden:** Conceptualization (lead); formal analysis (equal); methodology (equal); project administration (equal); supervision (equal); writing – review and editing (equal).

## CONFLICT OF INTEREST

The authors have stated explicitly that there are no conflicts of interest in connection with this article.

## ETHICS STATEMENT

The study was approved by the Newcastle University Faculty of Medical Sciences Research Ethics Committee (ref 1272/13714/2017) and informed consent was obtained from all participants. The study was performed in accordance with the Declaration of Helsinki.

## Supporting information

Supporting Information

## Data Availability

The data collected and analysed during the current study are available from the corresponding author on reasonable request.
